# Response-To-Intervention in Finland and the United States: Mathematics Learning Support as an Example

**DOI:** 10.3389/fpsyg.2018.00800

**Published:** 2018-06-05

**Authors:** Piia M. Björn, Mikko Aro, Tuire Koponen, Lynn S. Fuchs, Douglas Fuchs

**Affiliations:** ^1^School of Educational Sciences and Psychology, University of Eastern Finland, Joensuu, Finland; ^2^Department of Education, University of Jyväskylä, Jyväskylä, Finland; ^3^Department of Special Education, Peabody College, Vanderbilt University, Nashville, TN, United States

**Keywords:** comparative study, response to intervention framework, assessment, instruction, support in mathematics

## Abstract

Response to Intervention (RTI) was accepted in the early 2000s as a new framework for identifying learning difficulties (LD) in the U.S. In Finland, a similar multi-tiered framework has existed since 2010. In the present study, these frameworks are presented from the viewpoint of the role of assessment and instruction as expressed in documents that describe the frameworks, as it seems that these two components of RTI are the most disparate between the U.S. and Finland. We present a suggestion for the Finnish framework as an example of support in mathematics learning that incorporates principles of RTI (such as systematized assessment and instruction, cyclic support, and modifiable instruction). Finally, recommendations are presented for further refining and developing assessment and instruction policies in the two countries.

Why do we need educational frameworks and guidelines for providing support? Why can teachers not rely on their education and knowledge of learning and provide sufficient instruction and support for all students in need of something extra? These are the questions we discuss in the present paper. Different countries have different approaches to these matters, but we choose to compare the multi-tiered frameworks for support in learning used in the United States and Finland, as interesting similarities and dissimilarities exist. In the U.S., Response-To-Intervention (RTI) has long been a suggested framework for identifying students with disabilities. It provides guidelines for early prevention and for delivering evidence-based instruction with intensifying tiers of support. Close monitoring of student progress is also at the core of the U.S. RTI. Informed decision making at all levels within the system (administrative, teacher, and parental; see Fuchs and Fuchs, 2005) is provided. The basic idea of RTI in the U.S. is that the school provides the child with research-based instruction while the child is in the general education environment, and the school adjusts the intensity or nature of assessment and instruction according to the student's progress (Fuchs and Fuchs, 2005).

In our previous paper on the U.S. RTI and Finnish “RTI” (Björn et al., 2016), we found that the original purpose and, subsequently, the definition of RTI framework in these countries differed to some extent. The present paper on assessment and instruction within RTI frameworks in the U.S. and Finland is an extension of the previous papers. We previously found that RTI in the U.S. was primarily developed for LD (Learning Difficulty) identification, and the Finnish version was primarily intended to re-structure the existing support service framework for struggling students (Björn et al., 2016). Instead of the Finnish framework, the prevention of LD was an acknowledged goal in the frameworks of both countries. It seemed that the two frameworks were similar in appearance but differed in content and delivery. We wanted more knowledge that would explain why the renewed Finnish framework was outlined similar to the U.S. RTI, however, we found that the massive amount of existing knowledge on the pros and cons of the approach seemed to be neglected by the formal documents defining Finnish version of the framework, as many important definitions were not made explicit in formal documents.

We further realized that the role of the special education service system differed within the RTI framework in different parts of the U.S., while in Finland, special educational services have the same role within an RTI-like framework throughout the country (Björn et al., 2016). Thus, the present work presents the frameworks in the two countries but with a specific focus on assessment and instruction. The goal is to determine ways to refine both frameworks, with a special emphasis on bringing forward support for mathematics learning in Finland. We start this paper by briefly introducing the creation and implementation process of RTI in the U.S. and Finland. This is followed by differences and similarities in the definitions of assessment and instruction in these countries at each tier of support. Then, a suggestion for structuring support in mathematics in Finland is presented. We conclude by discussing possibilities for further refinements of the RTI approach in both countries.

## The creation and implementation of RTI in the U.S. and Finland

Some earlier studies have examined the differences between identification and learning support frameworks in the U.S. and Finland (see Itkonen and Jahnukainen, 2007; Jahnukainen, 2011; Björn et al., 2016). However, information about distinctions in the ways these countries operationalize RTI assessment and instruction is absent. The types of policy papers that present and compare educational frameworks implemented in different countries are important because even though the processes behind the reforms differ, the actual need for constructing frameworks for support in learning stems from the same source. That is, all education systems try to teach students effectively and at a reasonable cost. Such reforms are also nationwide processes, and each country may learn something from other countries despite cultural differences.

The U.S. school system consists of public and private schools. The average school age ranges from about age 5–18 years. The Finnish school system is public; there are basically no private schools. Children enter the compulsory schooling system the year they turn seven years old. Compulsory schooling lasts nine years, until the child reaches the age of 15 or 16 (depending on the time of year the child was born). The overall educational standards are run by the Ministry of Education, Science, and Culture, but the schools may relatively freely implement support in their own curricula (www.minedu.fi).

Although RTI in the U.S. as an approach to identifying and instructing especially students with LD has a long history (dating back to the 1970's), the implementation of RTI after the Individuals with Disabilities Education Act, (IDEA, 2004), enacted in 2004, has been interpreted as somewhat problematic. For example, Zirkel and Thomas (2010) conducted a survey that addressed the early years of RTI implementation in the U.S. Those authors found that although RTI has been an allowable substitute for the widely used IQ discrepancy criteria since 2004 (see Fuchs and Fuchs, 2006), confusion still persists between, for example, legal requirements and professional recommendations. Zirkel and Thomas have concluded that the legal content of RTI is still somewhat incomplete. This probably explains why countless versions of RTI have emerged. However, schools in the U.S. may still use the IQ discrepancy model along with RTI (see Zirkel, 2012a,b,c) in the process of identifying LD. Although RTI models vary considerably from state to state and from district to district in the U.S., many approaches are comparable to the three-tiered RTI framework currently in use in Finland (Björn et al., 2016).

In Finland, the Ministry of Education, Science, and Culture formed a steering group in 2006 to focus on developing a strategy for special education in basic education. Several tasks were to be achieved: developing ways to analyze the need for the amount of special educational services, developing legislation concerning special education, developing teacher education, developing administrative procedures in special educational services, and developing other areas related to special education. Consequently, a new strategy for special education was published in 2007.

Based on this strategy document, a renewed Basic Education Act was introduced in 2010 and was officially implemented in August 2011 in all Finnish schools (Pesonen et al., 2015). This lead to a framework with three levels of support for learning: Tier 1 general support (including co-teaching, differentiated teaching, etc. as forms of support); Tier 2 intensified support (domain-specific learning plans and support in reading, writingin flexible groups in addition to the forms of support mentioned before); and Tier 3 special support (all previous forms of support and individualized education plans) at each level, the student is entitled to a variety of forms of support (e.g., even special education, see Björn et al., 2016).

RTI as an approach to the identification and support of LD is gradually being implemented throughout Europe. For example, in the Netherlands, the Dutch Act on “Passend Onderwijs” adopted in 2014, states that all children should be included in mainstream education as much as possible, with financial support provided to schools by regional educational administrations. In addition to this, there is growing interest in using this framework throughout many countries in primary education (Scholvink and Janssen, 2014). According to the interpretation of RTI in the Netherlands, Tier 1 support is provided inside the classroom by the classroom teacher. This includes direct and differentiated instruction for all students. However, Tier 2 and Tier 3 support is mostly provided by a remedial teacher outside the classroom.

The U.S. RTI system has two main approaches to instruction: the problem-solving model and the standard protocol model (Fuchs et al., 2010; Jenkins et al., 2013). In the problem-solving model, a student's deficits are addressed by implementing a research-based intervention specially designed for that individual student (Johnson et al., 2006; Fuchs et al., 2010). Typically in the problem-solving model, decision-making teams, which may consist of teachers, administrators, school psychologists, and parents, follow a recursive four-step process: (a) define the problem, (b) plan an intervention, (c) implement the intervention, and (d) evaluate the student's progress (Fuchs et al., 2003; Bender and Shores, 2007). In the standard protocol model, students with similar difficulties (e.g., problems with reading fluency) are given research-based interventions that have been standardized and proven effective for students with similar difficulties for a predetermined amount of time (Johnson et al., 2006). The problem-solving model resembles the Finnish framework more than the standard protocol approach (Björn et al., 2016).

## RTI assessment and instruction

Because LDs (typically in reading or math or both) are major reasons for the need of extra support in learning (Fletcher et al., 2007), relevant guidelines for both assessment and instruction are needed (Fuchs et al., 2010, 2012). The concept of assessment is often viewed as unidirectional. It used to be interpreted as an authority administering assessments, with the examinee viewed as an object of classification (Ysseldyke et al., 1983). In RTI, however, as Grigorenko (2009) has noted, the roots of assessment in RTI seem to be related to dynamic assessment (DA; see also Elliott, 2000, 2003; Fuchs et al., 2007, 2011) in which the assessment is flexibly intertwined with teaching sequences. This enables up-to-date assessment results that can quickly inform the instruction. Relevant and supplementary skills-based testing is also an important component of RTI assessment as is progress monitoring. It has been proposed that the performance of “nonresponders,” (i.e., those children who do not show progress in academic skills) is monitored frequently with a set of short instruments relevant to these skills (Fuchs and Fuchs, 2005). By monitoring a student's learning and comparing it to that of peers receiving the same instruction, teachers can determine whether the student's academic level and rate of progress warrant further assessment or formal evaluation (Fuchs and Fuchs, 2005).

The first important assumption acknowledged in both RTI and DA is that conventional assessment does not work for children who have diverse educational and cultural experiences. These children are often those who need more intensified support in learning. The second assumption is that, instead of focusing on children's skills and abilities at a specific time (Fuchs et al., 2010), children have the potential to learn with adequate education or intervention (Fuchs et al., 2007). The third assumption is that the reason for assessment is to inform intervention, and consequently, the results of assessment should have direct implications for selecting or modifying instruction. The assessment data and continuous progress monitoring inform instruction at each tier. Additionally, research-based curriculum and instruction, as well as the systematic assessment of the fidelity with which instruction and interventions are implemented, are essential (National Association of State Directors of Special Education, 2005; Fuchs et al., 2007). It is important to note that assessment also includes other foci than learning outcomes in which the student's task-motivation (Eccles, 2005), academic self-efficacy, and metacognitive skills (Seaton et al., 2013) are taken into account in addition to the important assessment of the learning environment (Johnson et al., 2006).

Next, we will go through assessment and instruction policies in each overall Tier (the 3-tier RTI frameworks used here) comparing the US. and Finland. After that, we will present a model for providing individual support in mathematics according to Finnish RTI framework and legislation.

## Tier 1assessment and instruction

See Table 1 for a comparative presentation of assessment and instruction practices within RTI frameworks in the U.S. and Finland. Tier 1 in the U.S. RTI includes statewide norms as well as suggested materials and assessments usually performed within general education settings. On Tier 1, according to Fuchs and Fuchs (2005), struggling children are identified through poor performance in classwide, schoolwide, or districtwide screening intended to designate which children are at risk of academic or behavioral problems. In Finland, to date, there is *no formal guidance* on performing screenings within the RTI framework. Some type of universal screening might (once or twice per year), however, be performed according to a school's and municipality's own system. Finnish teachers may freely decide when, how, and with which the screenings are performed. The frequency of screening is normally three times per year in RTI, but once again, it is not clearly localized within the Finnish framework.

**Table 1:** Assessment and Instruction on each Tier of RTI/Level of support, Finnish framework.

	**RTI**	**Finnish framework**
	**Tier 1 (Primary prevention)**	**General support**
**ASSESSMENT**		
Type of assessment/identification	Universal Screening, statewide norms^a^	Not Specified (NS)^b^
Frequency of screening	3 times per year £	NS
Who does assessments	General education teacher	NS
Who makes decisions	Multiprofessional team, parents	Multiprofessional team, parents
Materials used in progress monitoring	CBM	NS, usually group assessments
Type of progress monitoring	Academic skills development monitoring	NS
Frequency of progress monitoring	Weekly, for 5-8 weeks for at-risk students	1–2 times per year
**INSTRUCTION**		
Length of Tier	max 8 weeks (1 year^∧^)	NS
Intensity of intervention(s)	90 min daily (in reading) £	Within regular school work, NS
Type of interventions	Core, With	NS/With, PT, SG, Ind
Type of instruction	Explicit, top-down (Differentiated instruction)	Differentiated instruction, etc.
Methods of interventions/instruction	Research-principled instruction, curricular^∧^	Flexible
Movement criteria between Tiers	Final status	NS
	**Tier 2 (Secondary prevention)**	**Intensified support**
**ASSESMENT**		
Type of assessment/identification	Instruction-based, skill-specific	NS
Who does assessments	Trained school personnel	School personnel
Who makes decisions	Multiprofessional teams, parents	Multiprofessional teams, parents
Materials used for assessments	Progress monitoring^c^	NS^c^
Type of progress monitoring	CBM	Learning plan assessment
Frequency of progress monitoring	No less than once every 2 weeks^∧^	NS
**INSTRUCTION**		
Length of Tier	max 1 school year (9-30 weeks^∧^)	NS
Intensity of intervention(s)	min 3 times/week, min 20–30 min/session^∧^	“More intense”
Type of interventions	Targeted/SG (3–5 students)	NS/PT, FT, With, SG, Ind
Type of instruction	Standard protocol, replicable (Team problem-solving, Behavioral consultation)	Flexible, NS
Methods of interventions/instruction	Specified programs, scripted protocols, evidence-b.	NS
Movement criteria between Tiers	Final status; cut point slope∞	NS
	**Tier 3 (Tertiary prevention)**	**Special support**
**ASSESSMENT**		
Type of assessment/identification	Curriculum-based, diagnostic	NS
Who does assessments	Highly skilled/educated school personnel	School personnel, consultation (medica
Who makes decisions	Multiprofessional teams, parents	Multiprofessional teams, parents
Materials used for assessments	Progress monitoring, diagnostic tools	Standardized tests available, but NS
Type of progress monitoring	CBM, diagnostic tests, IEP	Pedagogic plan assessment, IEP
Frequency of progress monitoring	No less than once a week^∧^	NS
**INSTRUCTION**		
Length of Tier	Min. 15–20 weeks^∧^	NS
Intensity of intervention(s)	More frequently than Tier 2, min 30 min/session^∧^	“More intense”
Type of interventions	Intense, SG, Ind (1–2 students)	Flexible, Ind, NS
Type of instruction	Data-based instruction (expert consultation)	PT, FT, With, SG, Ind
Methods of interventions/instructionSpecified programs, individual	Specified programs, individual	NS
Movement criteria between Tiers	Final status; cut point slope, individual progress^∧^	Re-assessments especially in transitions

a Screening, see: http://www.rti4success.org/screeningTools/

b *http://www.lukimat.fi/lukimat-oppimisen-arviointi/materiaalit/tuen-tarpeen-tunnistaminen: materials for performing universal screening exist but they are not formally linked to the renewed framework*.

c Progress monitoring, see: http://www.rti4success.org/progressMonitoringTools, https://charts.intensiveintervention.org/chart/progress-monitoring; Finnish progress monitoring would http://www.lukimat.fi/lukimat-oppimisen-arviointi/materiaalit/oppimisen-seuranta exist but they are not formally linked to the renewed framework; ^∧^Johnson, E., Mellard, D., Fuchs, D., McKnight, M. for NRCLD (2006); NS, Not Specified; PT, Part-time special education (in the USA: inclusive teaching); FT, Full-time special education (such as special classes, self-contained classrooms); With, Student within mainstream education, although has LD; SG, Small-group instruction (such as “Tier time,” resource rooms), Ind, Individual instruction.∞ as in performance below/above 25th percentile.£ These examples from New York State Special Education Department website: http://www.p12.nysed.gov/specialed/RTI/guidance/instruction.htm

The latest addition to the screening procedure in the U.S. RTI framework was suggested by Fuchs et al. (2012). Originally, support when moving from Tier 1 to Tier 2 was based on one screening phase according to which students who did not respond to instruction were referred for more intensive support. The new procedure involves a second stage of screening performed after a short period of support, which can contribute to accurate identification of students who require a supplemental layer of reading intervention (Compton et al., 2012) or math intervention (Fuchs et al., 2011). Another innovation by researchers actively working with the U.S. RTI was a second stage of diagnostic assessment that could be used to move students who did not respond to a supplemental layer of tutoring immediately to a more intensive and perhaps long-term intervention they required (Compton et al., 2012). Without such a second stage of screening, schools would provide costly intervention to many students who did not need it. Compton et al. (2012) have suggested a multistage screening process near the beginning of the first grade to avoid an “RTI wait-to-fail” model, in which children are required to participate in 10–30 weeks of supplemental intervention that could have been predicted to be inadequate.

In Finland, an *optional learning plan* is suggested (e.g., in the Basic Education Act, 2010) at the Tier 1 level called “general support.” This plan entails a means for assessment and support. The U.S. version of RTI suggests no such documentation. The frequency of progress monitoring (although it shows significant variation) is high within RTI and is not defined within the Finnish framework. In other words, in the renewed Finnish framework of support in learning, the role of assessment and instruction is somewhat undefined although the framework mentions possible forms of support (such as co-teaching, smaller study groups, etc.).

According to Fuchs and Fuchs (2005), in the three-tier U.S. RTI model, Tier 1 concerns at-risk children who have been identified through a screening process. They receive research-based instruction, sometimes in small groups, sometimes as part of a classwide intervention. A certain amount of time (generally not more than 6–8 weeks) is allotted to see if the child responds to the instruction. Each student's progress is monitored closely (for more information, see: http://www.rtinetwork.org/essential/assessment/progress/validated-forms-progressmonitoring). The intervention programs may be selected from a bank of research-proven interventions based on school resources in the U.S. The concept of progress monitoring (CBM) and a resource bank of suggested intervention methods are not mentioned at all in documents defining the Finnish framework.

## Tier 2 assessment and instruction

In the U.S. RTI, Tier 2 (also referred to as secondary prevention) belongs to general education as an instructional service. In Finland, this level called “intensified support,” including assessment as well as instruction, is organized via consultation and collaboration between teachers. In the U.S. RTI, assessment is instruction-based and skill-specific. The Finnish framework provides no formal guidance for assessment (in the sense of frequency). However, Finnish schools may, for example, decide whether to do a skill-specific assessment of students in need of extra support in learning. The Finnish framework provides for an *obligatory learning plan* at this level of intensified support in which the support a student receives is reported by teachers. No description of frequency or type of progress monitoring exists in the Finnish framework at the level of intensified support. The learning plan document consists of descriptions of different forms of support provided for a student. Large variation exists, as there is no guidance on time for support.

Multi-professional consultation is made in problem-solving RTI frameworks. Evidence-based protocols are used by reading specialists, special education teachers, and paraprofessionals in some RTI versions. Tier 2 within the RTI framework is an important stage between Tier 1 and the intensified Tier 3. Therefore, instruction on Tier 2 is evidence-based as well as performed in short periods to allow for the instruction to be modified in a timely manner (Fuchs and Fuchs, 2005). According to Fuchs and Fuchs (2005), if the child does not respond to the first level of group-oriented interventions, he or she typically is moved to the next RTI level. The length of time on Tier 2 has been reported to vary between 9 and 30 weeks, even one school year. The time allotted to see if the child responds to interventions at this more intensive level may be longer than on Tier 1. The intervention has been successful if the child shows adequate progress.

The group size of students receiving support given outside classrooms is another important feature on Tier 2 of RTI (Berkeley et al., 2009). For example, the state of Kansas has indicated that small-group instruction should consist of between three and five students on Tier 2 and fewer than three students on Tier 3. Other state models are more flexible in group size requirements. Arizona's model, for example, allows for large- or small-group instruction on Tier 1, small group instruction on Tier 2, and small or individualized instruction on Tier 3.

Within the Finnish framework, small-group instruction, along with the overall instruction that takes into account the diversity of students, is often described as “flexible.” This type of support is usually provided by special needs teachers or regular classroom teachers. However, co-teaching is a suggested form of support in the documents that have followed the actual Finnish law (for example, see Ahtiainen et al., 2012).

## Tier 3 assessment and instruction

Tier 3 in the U.S. framework differs in many ways from the equivalent level of the Finnish framework, which is called “special support.” For example, the RTI framework in many US states does not include any form of special education at this tier (although it has been frequently suggested by researchers in the field, see the work of Fuchs and Fuchs, 2005, for example). In contrast, this tier entirely belongs to special education in Finland although a student might still receive support and instruction in regular classroom instruction. If the support offered within the first two RTI tiers in the US has not been enough, significantly more intensified (no less than once a week for 15–20 weeks) instruction is then essential (Fuchs and Fuchs, 2005). Furthermore, if the child does not respond to instruction at this level, then he or she is likely to be referred for a full and individual evaluation. This referral is a major difference between the U.S. version of RTI and the Finnish framework. The child has already been assessed many times during the level of intensified support in Finland, but not in a unified manner across municipalities or schools within the same district as there is a lack in formal guidance on performing the assessment. Access to special education services in Finland does not require statements of eligibility but is based on multidisciplinary decision-making that also involves the caregivers' opinions. The U.S. RTI provides for instruction for one or two students at a time. The Finnish system lacks explicit min–max descriptions for different levels of support, but many times a student on Tier 3 is situated, at least part of a day, in small groups outside the regular classroom for the most important content areas (usually literacy skills and mathematics). All possible forms of instruction are in use at this level of support in Finland. An obligatory pedagogical review is conducted of all students, and the existing means of support and goals for learning are defined in this review.

Tier 3 RTI in the U.S. has an interesting feature: individualized data-based instruction (or experimental teaching; for a case example, see Fuchs et al., 2010). DBI is a research-based process for individualizing and intensifying interventions through the systematic use of assessment data, validated interventions, and research-based adaptation strategies (see more at: http://www.intensiveintervention.org). This form of instruction resembles in many ways the flexibility and degree of individual assessment and instruction that exists in the Finnish framework; teaching methods are individually adjusted. However, what is missing from the background of the Finnish framework is a research-based resource center that would actually validate using individually adjusted instructional methods.

Assessment and instruction in the U.S. RTI framework seem to be closely intertwined. First, the forms of assessment are defined in more detail in the U.S. framework. Second, the main forms of instruction/intervention delivered to students within the U.S. RTI framework rely on research-based interventions, which often include well-defined assessment and programmatic content designed to ensure intensity and duration (Fuchs and Fuchs, 2006). In contrast, the Finnish framework does not include clear definitions for support or follow-up of learning results. Because students with severe learning difficulties in mathematics are in need of the most intensive support, we will next present a suggestion for refining the Finnish framework in terms of individual support in mathematics. Note that our suggestion might be used in other content areas as well.

## Finnish framework for individual support in mathematics: an example of RTI interpretation

We have identified a national need for bringing more content and research-based substance to the RTI-like framework, as well as a more systematized approach in Finland. This can be done by providing the support stipulated in formal legislation and other documents schools and teachers currently use in their everyday work. We have not tried to present everything as so much better in the U.S, by using U.S. RTI as an example, but we want to point out that the way Finnish three-tiered framework is currently presented has left too much room for local interpretation. By discussing this in an international forum, we believe that other countries currently in the process of developing their own RTI frameworks might be able to handle building and implementing the framework even better than Finland and the U.S.

We have published a more comprehensive Finnish version of this suggestion on support in mathematics (see Björn et al., 2015) incorporating all three tiers of support, but we have rethought and refined the model in terms of Tier 3 support in mathematics for the present paper. Overall, our suggestion needs to omit some of the principles already in use in the U.S., but that is mainly due to the current lack of material (e.g., assessment tools, progress monitoring tools, etc.) Our suggestion follows Finnish legislation and the outline of the Finnish RTI or “three-tiered framework for support” but incorporates the suggestions of Gersten et al. (2009) and Bryant et al. (2014).

Slavin and Lake (2008) have pointed out that the best learning results in mathematics may be achieved by using systematized, yet flexible, ways of support. Which means that teachers should be given possibilities to modify the support offered (see, Lemons et al., 2014). In Finland, special educational services (as in support provided by a special needs teacher) are available at all three tiers of support. However, the main principle should be that the more intensive the need for support, the more individualized support should be given (Gersten et al., 2009). Consequently, Tier 3 support in primary school should mean choosing evidence-based intervention material as the basis for planning mathematics instruction (Mononen, 2014).

Tier 1 and Tier 2 support precede Tier 3. If preliminary support for learning mathematics in the classroom as part of a large group or even occasionally as part of a small group had been attempted without clear signs of acceleration of math skills, then, according to Finnish law, a formal referral to special education would be needed for Tier 3 support. Subsequently, an individual education plan (IEP) with plans for instruction would be drawn up with the participation of the student, caregivers, school psychologist, classroom teacher, and special needs teacher. We suggest that approach to instruction during a school year would consist of several cycles. The current situation in schools is that each teacher (or teacher and special needs teacher-pair) decide on the frequency and content of support. This results in differing practices, and the rights of suitable instruction provided for each individual student in need of support in mathematics are not addressed adequately.

To correct this situation, we suggest that each cycle of support lasts for 5–7 weeks, and that the support is provided 3–4 times per week (each session duration 30 min of intensive work). starting from making sure very basic math skills are learned (number line skills backwards and onwards, calculations including additions, subtractions, overall estimation ability). By viewing the support as cycles throughout the school year, groups/pairs of students participating in Tier 3 support could work together regardless of age. We recommend that students work in pairs or small groups, maybe occasionally even provided with fully individualized support. This means that based on “what works” literature (see Gersten et al., 2009), students that need intensive support in mathematics would benefit the most from support given in smaller groups rather than in a large classroom group. We have conducted an intervention with trials on individual support provided in a regular classroom for students in need of support in mathematics Björn et al., (under review), but those inclusion trials did not fully convince us of their superiority to small-group intervention outside the regular classroom. Consequently, we think that intensive intervention periods provided as relatively short cycles, instead of continuous intensive instruction, would enable testing the regular classroom as a learning environment occasionally, and, if sufficient skills have been learned, the “pull out” type of instruction/intervention outside the regular classroom could be stopped at some point.

Each support cycle would begin and end with a short assessment of learning gains so that adjustments of instruction could be made in a timely manner. A cyclic assessment also enables the teacher to determine the point at which each basic math skill has been learned. This way, the approach for assessment and instruction would be “continuous” in terms of what we know about the persistence of developmental mathematics learning difficulties (Fletcher et al., 2007).

We cannot expect severe mathematics learning difficulties to be “cured” even by repeating several cycles of support during a school year; instead, support would need to be provided over several school years. The teaching contents during these support sessions would include basic arithmetic and estimation skills, according to individual needs, for as long as deemed necessary. Continuity of the support would be ensured by keeping a record of the support and assessment given to each student. Giving many alternate suggestions for intervention programs to be selected from as the instructional basis for this support cannot be done at this time. This is due to the fact that, to date, only a few intervention programs for mathematics are available in Finland (for more information, see www.lukimat.fi).

What we have presented here can be summed up like this; assessment and instruction on Tier 3 (special support) should be continuous, cyclic, individual, and based on evidence-based intervention programs. Support can be provided in many different contexts, but it must be systematized and modifiable between cycles.

## Discussion

In this paper, we presented the RTI framework and the three-level Finnish educational support system from the viewpoints of assessment and instruction. The models were implemented based on similar background philosophies: the right to receive the best possible preventive support for learning and participation. Tohe recent Finnish reform (Basic Education Act, 2010), after many phases, developed into a model in similar to the U.S. RTI model (Fuchs and Fuchs, 2005), at least on the surface. However, there are many differences that might give new insights to any country planning to develop similar frameworks. For example, the current U.S. model aims for the identification and prevention of further learning difficulties (Compton et al., 2012) by placing a student within a suitable tier of intervention (Vaughn and Fuchs, 2003). The Finnish model, in contrast, mainly aims at supporting learning at the earliest time point possible (Opetusministeriö. Erityisopetuksen strategia, 2007) within the three-tiered framework.

The major finding of the present analysis is that unlike the renewed Finnish system of support in education, the U.S. RTI framework included as early as 2004 many suggested materials for universal screening, early intervention, multi-tiered levels of support, evidence-based intervention, data-based decision-making regarding intervention, and using students' responsiveness to evidence-based instruction in evaluating disability status (Haager et al., 2007). RTI in the U.S. has succeeded in accelerating a paradigmatic change in the uses of testing. Instead of focusing on learning achievement at one point, RTI focuses on individual responses in relation to instruction (Fletcher and Vaughn, 2009; Fuchs et al., 2010).

Moreover, the concept of evidence-based teaching or evidence-based intervention is not present in either of the Finnish documents (Opetusministeriö. Erityisopetuksen strategia, 2007; Basic Education Act, 2010) or in Finnish schools. In the Finnish model, individual assessment (progress when receiving support) is not described. Thus, one major observation that might explain why there is such a noticeable difference between RTI and the Finnish framework is that there is no such large degree of teacher accountability in Finnish school culture (see Sahlberg, 2010) as may be observed to exist within the U.S. For example, the concept of “fidelity to instruction” (Fuchs et al., 2007) is not yet in use in Finland. Instead, the concept of “trust” is used frequently (see Itkonen and Jahnukainen, 2007) when talking about teachers' work.

Municipalities, schools, and teachers in Finland have a relatively broad autonomy in interpreting legislation and curricular instructions. One reason for this is the equity of the Finnish educational policy system (Linnakylä et al., 2011). Another reason for this type of freedom is that Finnish teachers must have a Master's degree in education to be recruited to a permanent teaching position. Due to this high educational level, Finnish teachers are often deemed as trusted professionals. Therefore, they are used to making decisions on how to assess students' skills, what type of instruction to apply, and how long to give instruction before making a decision on whether or not to move the student to the next level of support. This results in very individual and different ways of supporting students' learning processes. However, bringing a more interventionist approach to learning support within the Finnish educational system would allow more systematic development of instructional practices as well as accumulation, documentation, and distribution of knowledge. Also clear instructions on how to implement these practices are still needed. That is why we have presented a suggestion for providing support in mathematics. However, we are well aware that this suggestion will not be taken seriously as long as the formal documents praise the pedagogical freedom of teachers and local solutions (as suggested in problem-solving RTI models) for learning difficulties.

A debate about the aims, justification, and uses of the framework of the U.S. RTI (e.g., Artiles et al., 2010; Fuchs et al., 2010; Vaughn et al., 2010; Fuchs and Fuchs, 2017) is still ongoing. Perhaps one way to further clarify the uses of RTI in the US would be that, because they are originally based on the traditions of dynamic pairing of assessment and instruction, they should be seen as a series of carefully selected protocols in the future (Fuchs et al., 2010). This would ensure instructional replicability and flexibility, and the process of identifying learning difficulties would be made clearer.

## Recommendations

Within education systems there are always possibilities for improvement, even after reforms take effect. The present analysis contributes to this goal and to the research literature by identifying similarities and differences between two countries with significant experience of RTI-like frameworks. Because formal identification of learning disabilities is not a central part of the current Finnish framework, it is understandable that it resembles those RTI systems that take a problem-solving and consultation-based approach (Ikeda and Gustafson, 2002). A much-welcomed addition to the Finnish RTI would be the data-informed, decision-making and systemized use of standardized assessment and instruction tools, based on systematized progress monitoring (see Fuchs and Fuchs, 2005). This is a question of the allocation of funds that have not been directed toward developing assessment tools and intervention programs in Finland. This is a major difference between Finland and the U.S., where major technical assistance centers, with federal funding, are available to support RTI implementation (e.g., the National Center for Response to Intervention (2016; http://www.rti4success.org/); the National Center for Intensive Intervention (National Center for Intensive Intervention; http://www.intensiveintervention.org).

If Finland would like to move toward evidence-based or research-based instruction in schools, one of the existing stakeholders (e.g., the Finnish National Board of Education or the Ministry of Culture and Education) should take steps toward establishing similar centers. However, we continuously seek funding to make the www.lukimat.fi service a national RTI center that would be strongly connected to the best universities with the aim of developing evidence-based intervention and advising teachers in addressing learning difficulties.

Although the RTI framework seems to be clear, the IDEA legislation leaves too much room for multifaceted interpretations, a situation that leads to, for example, seven-tiered RTI models and the impossibility of comparing the uses of RTI across the U.S. On the other hand, the three-tiered Finnish framework is clear in its background philosophy and purpose (Sabel et al., 2011), but it lacks content: no assessment or intervention tools have been indicated although there are a few available. This lack of indication of materials has led to multiple interpretations of what qualifies as assessment tools (and to discussions if there is a need for using assessment tools at all) and of what intensified or special instruction means.

## Conclusions

What follows from revealing these differing profiles of assessment and instruction within the two countries are some modest suggestions for concluding remarks. For the RTI model used in the U.S., it would be useful to simplify the RTI models in use (see also, Fuchs and Fuchs, 2017) and return to its origins: a three-tiered model with research-based instruction on the first tier, standard protocols on the second tier, and intensive, method-rich, research-based teaching on the third tier. With regards to the future of the Finnish model, the priority, of course, is to collect and create a national resource for assessment materials as well as intervention materials suitable for instructional packages with different intensities and lengths. This process would lead to the use of similar assessment methods and intensified instruction across schools and municipalities and also cumulative knowledge on “what works with whom.” Because the current legislative framework in Finland clearly indicates that support for learning with increasing intensity is required by law, now is a good time to start developing actual assessment policies and ways to implement evidence-based instruction practices intended for the support of learning.

## Author contributions

PB has been the major contributor of this paper. LF and DF have provided information on the U.S. RTI. TK and MA have reviewed the paper several times, commented and contributed to the discussion.

### Conflict of interest statement

The authors declare that the research was conducted in the absence of any commercial or financial relationships that could be construed as a potential conflict of interest.
